# Identification of Potential Biomarkers of Septic Shock Based on Pathway and Transcriptome Analyses of Immune-Related Genes

**DOI:** 10.1155/2023/9991613

**Published:** 2023-08-05

**Authors:** Jie Wang, Jie Cai, Linlin Yue, Xixi Zhou, Chunlin Hu, Hongquan Zhu

**Affiliations:** ^1^Department of Critical Care Medicine, The First Affiliated Hospital of Gannan Medical University, Ganzhou, Jiangxi 341000, China; ^2^Department of Critical Care Medicine, HUST Union Shenzhen Hospital (Nanshan Hospital), Shenzhen, Guangdong 518052, China; ^3^Department of Emergency Medicine, The First Affiliated Hospital of Sun Yat-sen University, Guangzhou, 510080 Guangdong, China

## Abstract

Immunoregulation is crucial to septic shock (SS) but has not been clearly explained. Our aim was to explore potential biomarkers for SS by pathway and transcriptional analyses of immune-related genes to improve early detection. GSE57065 and GSE95233 microarray data were used to screen differentially expressed genes (DEGs) in SS. Gene Ontology and KEGG (Kyoto Encyclopedia of Genes and Genomes) pathway enrichment analyses of DEGs were performed, and correlations between immune cell and pathway enrichment scores were analyzed. The predictive value of candidate genes was evaluated by receiver operating characteristic (ROC) curves. GSE66099, GSE4607, and GSE13904 datasets were used for external validation. Blood samples from six patients and six controls were collected for validation by qRT-PCR and western blotting. In total, 550 DEGs in SS were identified; these genes were involved in the immune response, inflammation, and infection. Immune-related pathways and levels of infiltration of CD4 + TCM, CD8 + T cells, and preadipocytes differed between SS cases and controls. Seventeen genes were identified as potential biomarkers of SS (areas under ROC curves >0.9). The downregulation of *CD8A*, *CD247*, *CD3G*, *LCK*, and *HLA-DRA* in SS was experimentally confirmed. We identified several immune-related biomarkers in SS that may improve early identification of disease risk.

## 1. Introduction

Septic shock (SS) is defined as an infection-related circulatory dysfunction and metabolic disorder and is clinically associated with myocardial dysfunction and decreased ejection fraction [1, 2]. Patient signs and symptoms include fever, rapid heart rate, shortness of breath, weakness and sweating, hypoxia, and altered mental status [3]. Kidney failure, malignancy, diabetes, chronic lung disease, congestive heart failure, and immunosuppression may also increase the risk of SS [4]. Currently, SS is the leading cause of death among hospitalized patients, with a fatality rate of up to 40% [5, 6]. Although tissue perfusion can be quickly regained in patients with SS after a positive fluid resuscitation and symptomatic treatment with vasoactive drugs and anti-infection agents, the risk of recurrence after hospital discharge is high [7]. Furthermore, more than half of the survivors have impaired physical or neurocognitive function, mood disorders, and poor quality of life [8, 9]. To reduce the mortality rate and improve the quality of life of patients after SS resuscitation, clinical studies are ongoing; however, early untreatable multiorgan failure makes the recovery process difficult. Therefore, in addition to improving programmed management, increasing the specificity of early recognition can also effectively improve survival in SS.

Innate immunity and adaptive immunity play a key role in the response to SS. During the induced inflammatory response, monocytes, macrophages, and neutrophils are activated, exacerbating vascular damage by producing cytokines, proteases, kinases, and reactive oxygen species [10]. Immunosuppressive responses are active in patients with SS [11], and the apoptosis of B cells and follicular dendritic cells is involved in this process [12]. T lymphocytes contribute markedly to the immune system, and T-cell abnormalities have been found in patients with SS [13]. Furthermore, immune dysfunction has been shown to impair the ability to clear primary infections and increase secondary infections [14, 15]. Tolsma et al. found that neutropenia and specific immunodeficiency are independently associated with an increased risk of death in patients with SS [16]. However, the mechanism by which immune dysfunction contributes to the progression of SS remains unclear despite the important implications for the development of biomarkers.

In this study, we analyzed the immune-related pathways involved in SS progression and identified candidate biomarkers. First, we screened differentially expressed genes (DEGs) between patients with SS and healthy controls based on gene expression data from public databases, followed by enrichment analyses of DEGs. An immune cell deconvolution analysis and pathway-immune cell correlation analysis were performed to identify key immune-related genes. Finally, the expression of key genes was validated by quantitative real-time polymerase chain reaction (qRT-PCR) and western blotting. A flowchart of the study is displayed in Figure S1. Our findings are expected to contribute to the development of personalized immunotherapy regimens for patients with SS.

## 2. Materials and Methods

### 2.1. Data Acquisition

Two microarray datasets, GSE57065 and GSE95233, were downloaded from the Gene Expression Omnibus (GEO, http://www.ncbi.nlm.nih.gov/geo/) [17]. GSE57065 comprised data for 28 patients whose blood samples were collected within 30 min, 24 h, and 48 h after SS and 25 healthy controls. The GSE95233 included 22 healthy controls and 51 patients with SS, sampled twice at admission and once at D2 or D3. All samples from the GSE57065 and GSE95233 datasets were detected using the GPL570 [HG-U133_Plus_2] Affymetrix Human Genome U133 Plus 2.0 Array. GSE4607 and GSE13904 were used for the external expression validation of candidate genes between healthy controls and SS. GSE66099, which includes data for 30 systemic inflammatory response syndrome (SIRS) and 181 SS samples, was used to compare the expression differences between SS samples and the samples of the diseases with similar or related phenotypes to SS.

### 2.2. Data Preprocessing and Gene Annotation

Based on the two datasets, the probe expression matrix after normalization and log2 transformation was downloaded. Thereafter, the annotation file from the detection platform was obtained to match the gene symbol by the probe number inside; probes that did not match a gene symbol were removed. If different probes were mapped to the same gene symbol, the mean value of the probes was used as the final expression value for the gene.

### 2.3. Screening for DEGs between SS Samples and Controls

The limma package version 3.10.3 in *R* [18] (http://www.bioconductor.org/packages/2.9/bioc/html/limma.html) was used to analyze differences in gene expression between SS samples and healthy controls. The Benjamini & Hochberg (BH) method [19] was used to calculate the adjusted *p* value. Genes with an adjusted *p* value <0.05 and |logfold change (FC)| > 1 were selected as DEGs.

### 2.4. Functional Enrichment Analyses of DEGs

DAVID version 6.8 [20] (https://david-d.ncifcrf.gov/) was applied for Gene Ontology (GO) [21] enrichment analysis of DEGs according to three main categories, biological process (BP), cell component (CC), molecular function (MF), as well as Kyoto Encyclopedia of Genes and Genomes (KEGG) [22] pathway enrichment analyses. Results with *p* < 0.05 and gene count >2 were considered statistically significant.

### 2.5. Protein–Protein Interaction (PPI) Network Construction

The intersecting DEGs between the GSE57065 and GSE95233 datasets were obtained to predict the PPI using STRING version 10.0 [23] (http://www.string-db.org/). During this analysis, the species was set to *Homo*, and the highest confidence score was set to 0.9. Cytoscape version 3.4.0 [24] (http://chianti.ucsd.edu/cytoscape-3.4.0/) was used to visualize the PPI network, and CytoNCA plug-in version 2.1.6 [25] (http://apps.cytoscape.org/apps/cytonca) was used to analyze the node degree with parameter setting “without weight.”

### 2.6. Gene Set Enrichment Analysis (GSEA)

The *R* package clusterProfiler version 3.16.0 [26] (http://bioconductor.org/packages/release/bioc/html/clusterProfiler.html) was used to perform GSEA. The KEGG pathway gene set in the molecular signature database [27] (MSigDB, http://software.broadinstitute.org/gsea/msigdb/index.jsp) was used as the background gene set. DEGs between SS samples and controls with adjusted *p* < 0.05 and |logFC| > 0.263 were selected as input genes and were sorted in descending order by logFC. BH was finally used to adjust *p* values, and pathways with adjusted *p* values less than 0.05 were considered statistically significant.

### 2.7. Differential Analysis of Functions and Pathways

By considering c2.cp.kegg.v7.1.symbols.gmt in MSigDB as the background gene set, the *R* package GSVA version 1.36.2 [28] (http://bioconductor.org/packages/release/bioc/html/GSVA.html) was used to calculate the enrichment scores for KEGG pathways in each sample. A score matrix was obtained. Significant KEGG pathway differences were identified using the limma package with threshold values of adjusted *p* value <0.05 and |logFC| > 1.

### 2.8. Immune and Stromal Cell Deconvolution Analysis

xCell [29] (https://xcell.ucsf.edu/) was used to estimate the enrichment scores for 64 types of immune and stromal cells with a threshold of *p* < 0.05. The Wilcoxon test was then used to compare enrichment scores between SS samples and controls, and significance was set at *p* < 0.05. The shared differentially enriched immune cells between GSE57065 and GSE95233 were obtained as candidates for further analyses.

### 2.9. Analysis of Correlations between Pathways and Immune Cells

By considering the intersection of results obtained by GSEA and GSVA, key pathways were selected. Spearman correlation coefficients for relationships between the pathway enrichment score and immune cell deconvolution score in each sample were computed. The intersecting relations with adjusted *p* value <0.05 and |*r*| > 0.4 for GSE57065 and GSE95233 were selected as significant cell-pathway interactions.

### 2.10. Evaluation of Key Genes

The intersection of pathway-related genes involved in cell-pathway interactions and genes in the PPI network with degrees over 10 was identified as key genes. The *R* package ggstatsplot version 0.5.0 was used to construct violin plots of key genes in different groups, while the *R* package plotROC version 2.2.1 was used to generate the diagnostic receiver operator characteristic (ROC) curve for each gene.

### 2.11. Blood Sample Collection

Whole blood samples and white blood cell samples from 12 participants (six patients with SS and six healthy controls) were collected for qRT-PCR and western blotting, respectively. Patients were diagnosed according to the criteria defined by the European Society of Intensive Care Medicine/Society of Critical Care Medicine. Patients with SS required a vasopressor to maintain a mean arterial pressure greater than 65 mmHg and elevated serum lactate greater than 2 mmol/L, despite adequate fluid resuscitation [6, 30]. Patients with cancer, diabetes, autoimmune diseases, or a history of viral infection were excluded from the study. Healthy volunteers were enrolled from the physical examination center, and subjects with a history of major disease or infection were excluded. All subjects were informed of the aims and procedures of this study, and signed informed consent was obtained accordingly. This study was approved by the Ethics Committee of the First Affiliated Hospital of Gannan Medical University (No : LLSC-2021101302) and conformed with the Declaration of Helsinki.

### 2.12. qRT-PCR Analysis

The core genes in the PPI network and genes with larger fold change values were selected for qRT-PCR. Total RNA was extracted from whole blood samples using TRIzol reagent (9109; TaKaRa, Kusatsu). After reverse transcription, cDNA was quantified by real-time PCR with the ABI 7900HT FAST (Applied Biosystems) and Power SYBR Green PCR Master Mix Kit (A25742; Thermo, Waltham, MA, USA). *GAPDH* was used as an internal reference. The forward and reverse primer sequences are shown in Table S1. The reaction conditions were as follows: 50.0°C for 2 min, 95.0°C for 10 min, and 40 cycles at 95.0°C for 15 s and 60.0°C for 60 s. The relative expression level was detected in triplicate and was normalized using the 2^−ΔΔct^ method.

### 2.13. Western Blot Analysis

Genes with the largest degree of connections in the PPI network were selected for western blot analysis. Antibodies against HLA-DRA (A11787) and LCK (A2177) were obtained from ABclonal (Wuhan, China), while the GAPDH antibody (60004-1-lg) was purchased from Proteintech (Rosemont, IL, USA). Western blot analysis was performed using standard procedures with anti-HLA-DRA (1 : 1000) and anti-LCK (1 : 1000). After 2 h of incubation with secondary antibodies (anti-rabbit IgG: 111-035-045, Jackson; anti-mouse IgG: 115-035-003, Jackson), the protein bands were developed via chemiluminescence using the Millipore ECL system (Billerica, MA, USA). Thereafter, the bands were scanned and recorded using Tanon Image (Tanon, Shanghai, China).

### 2.14. Statistical Analysis

Tanon Image was used to analyze the gray level of the western blot data. The qRT-PCR and western blot results were analyzed using GraphPad Prism 5 (GraphPad Software, San Diego, CA, USA) and visualized using histograms. Differences in the mRNA and protein expression levels of candidate genes between the SS and control groups were analyzed by *t*-tests. Statistics *p* < 0.05, *p* < 0.01, and *p* < 0.001 represent significant, highly significant, and extremely significant, respectively.

## 3. Results

### 3.1. Screening of DEGs between SS Samples and Healthy Controls

**Figure 1:**
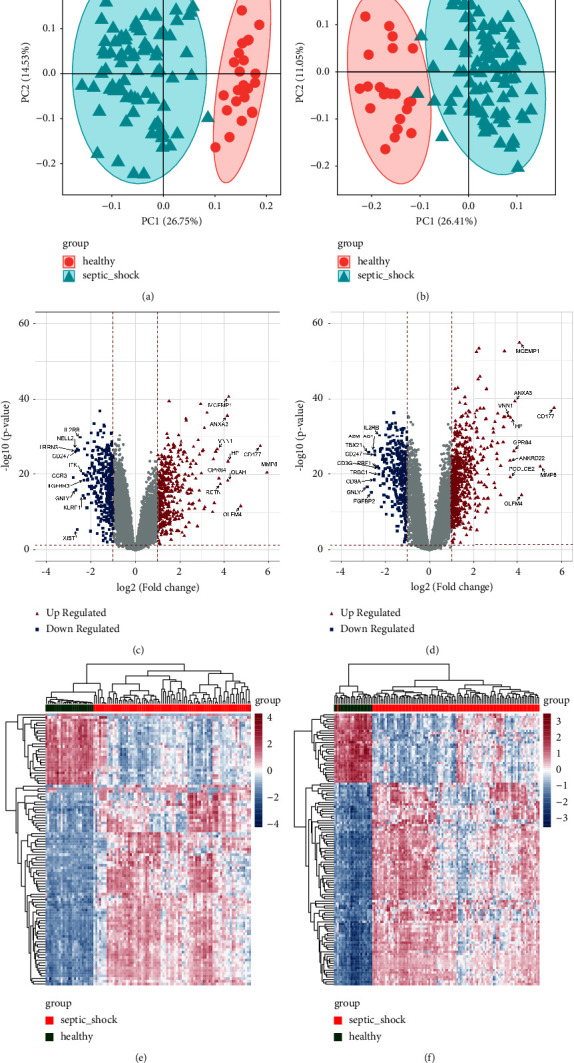
Screening of DEGs between SS samples and controls. PCA diagrams of samples in GSE57065 (a) and GSE95233 (b) suggest that SS samples and control samples could be clearly distinguished, with no significant outliers. A total of 763 and 933 DEGs from GSE57065 (c) and GSE95233 (d) were detected. Red triangles and blue squares represent upregulated and downregulated DEGs, respectively. The top 10 upregulated and downregulated DEGs based on logFC values are labeled. Heatmaps showing the expression differences of DEGs with |logFC| > 2 between SS samples and controls in GSE57065 (e) and GSE95233 (f).

Based on the expression matrix of GSE57065 and GSE95233, principal component analysis (PCA) was performed (Figures 1(a) and 1(b)), which revealed a sharp distinction between the SS and control groups in both datasets. There were no significant outliers, and all samples could be used for further analyses. In total, 763 and 933 DEGs were obtained from GSE57065 and GSE95233, respectively, as shown in Figures 1(c) and 1(d). DEGs with |logFC| > 2 were selected to construct a heatmap (Figures 1(e) and 1(f)); these genes were identified as significantly differentially expressed between the SS and control groups.

### 3.2. Function and Pathway Enrichment Analyses of DEGs

**Figure 2:**
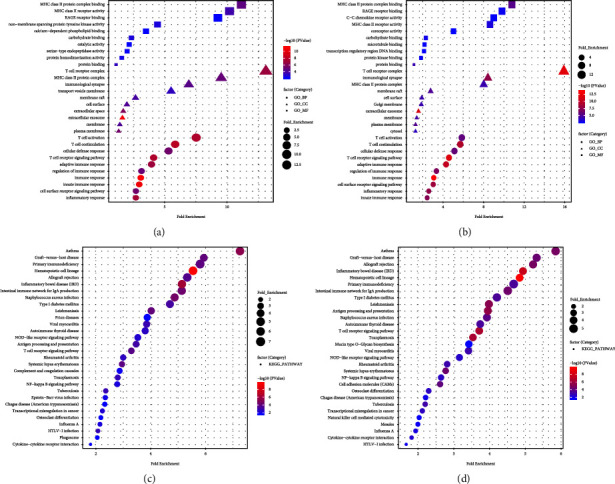
Functional and pathway enrichment analyses of DEGs. Top 10 GO functions of DEGs in GSE57065 (a) and GSE95233 (b) in the BP, CC, and MF categories. Enriched KEGG pathways for GSE57065 (c) and GSE95233 (d). The x-axis indicates fold enrichment, and the y-axis indicates GO functions and KEGG pathways. The larger the bubble, the greater the fold enrichment. The smaller the bubble, the smaller the *p* value.

GO and KEGG pathway enrichment analyses of the DEGs in the two datasets were performed. DEGs in GSE57065 were mainly enriched for 124 GO-BP terms, 34 GO-CC terms, 30 GO-MF terms, and 32 KEGG pathways. DEGs in GSE95233 were mainly enriched for 127 GO-BP terms, 37 GO-CC terms, 41 GO-MF terms, and 34 KEGG pathways. Based on the ranking of *p* values, the top 10 GO functions for GSE57065 and GSE95233 are displayed in Figures 2(a) and 2(b). Among them, the MHC class II protein complex binding of GO-MF, T-cell receptor complex of GO-CC, and T-cell activation of GO-BP showed the highest fold enrichment in both datasets. The enriched KEGG pathways were broadly consistent across the two datasets (Figures 2(c) and 2(d)), thereby suggesting good homogeneity and reliability among GSE57065 and GSE95233.

### 3.3. Construction of a PPI Network

**Figure 3:**
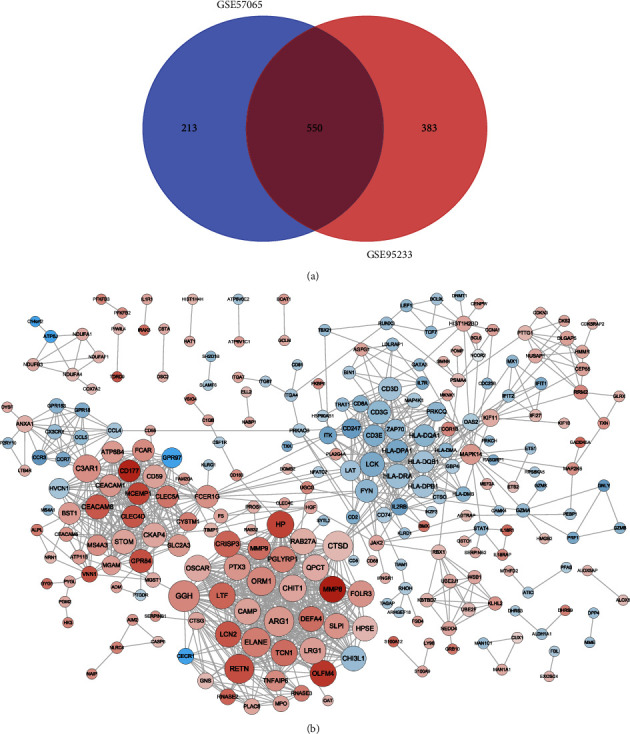
PPI network construction. (a) Venn diagram showing 550 intersecting DEGs in the GSE57065 and GSE95233 datasets. (b) PPI network involving 243 DEGs. Red and green circles represent upregulated and downregulated DEGs, respectively. The larger the node, the greater the degree. Gray lines represent protein-protein interactions. The darker the line, the larger the value of |logFC|.

The intersection of DEGs in GSE57065 and GSE95233 included 550 genes (Figure 3(a)). These DEGs were used to construct a PPI using STRING. A total of 1,104 interactions involving 243 proteins were obtained (Figure 3(b)). Nodes with more connections had larger contributions to the PPI network.

### 3.4. GSEA

**Figure 4:**
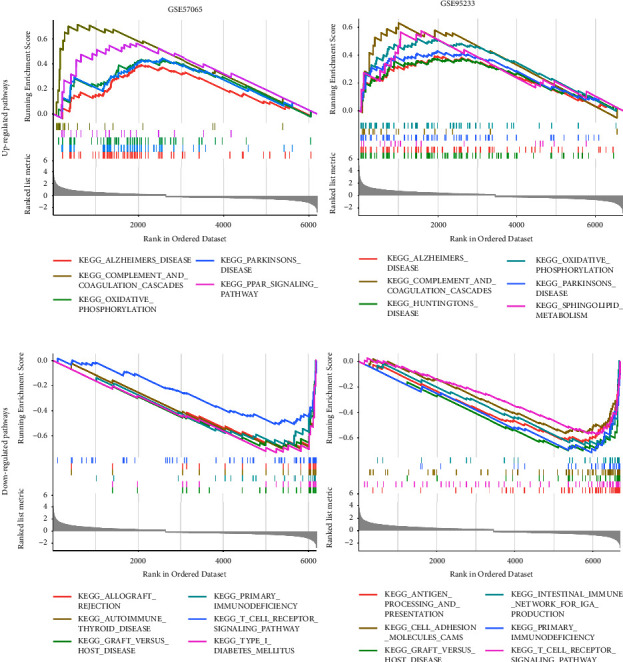
GSEA. Top 6 upregulated and downregulated pathways in GSE57065 and GSE95233 according to NES ranking.

With GSEA, 5 upregulated and 13 downregulated pathways were obtained in GSE57065, whereas 7 upregulated and 15 downregulated pathways were obtained in GSE95233. According to the normalized enrichment scores (NES), the top six upregulated pathways, including Alzheimer's disease, complement and coagulation cascades, oxidative phosphorylation, and Parkinson's disease, and the top six downregulated pathways, such as graft versus host disease, primary immunodeficiency, and T-cell receptor signaling pathway, were selected in both datasets and are displayed in Figure 4.

### 3.5. Differential Analysis of Enriched Pathways

**Figure 5:**
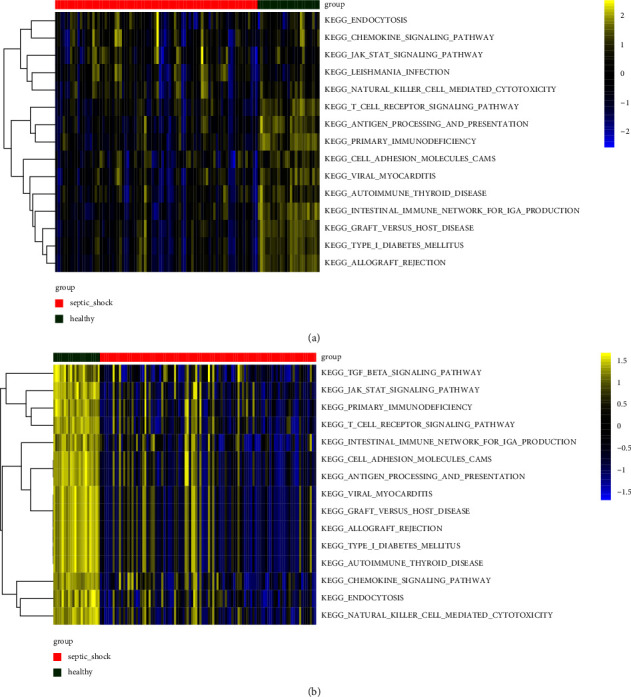
Differential analysis of enriched pathways in the two datasets. Heatmaps show KEGG pathways with significant differences in enrichment scores between SS and control samples in GSE57065 (a) and GSE95233 (b).

The enrichment scores for KEGG pathways for each sample were calculated using the GSVA algorithm. Based on the differential analysis of enriched pathways, both GSE57065 and GSE95233 had 15 significantly downregulated KEGG pathways. As shown in heatmaps (Figures 5(a) and 5(b)), enrichment scores for these pathways differed significantly between the SS and control groups.

### 3.6. Deconvolution Analysis of Immune and Stromal Cells

**Figure 6:**
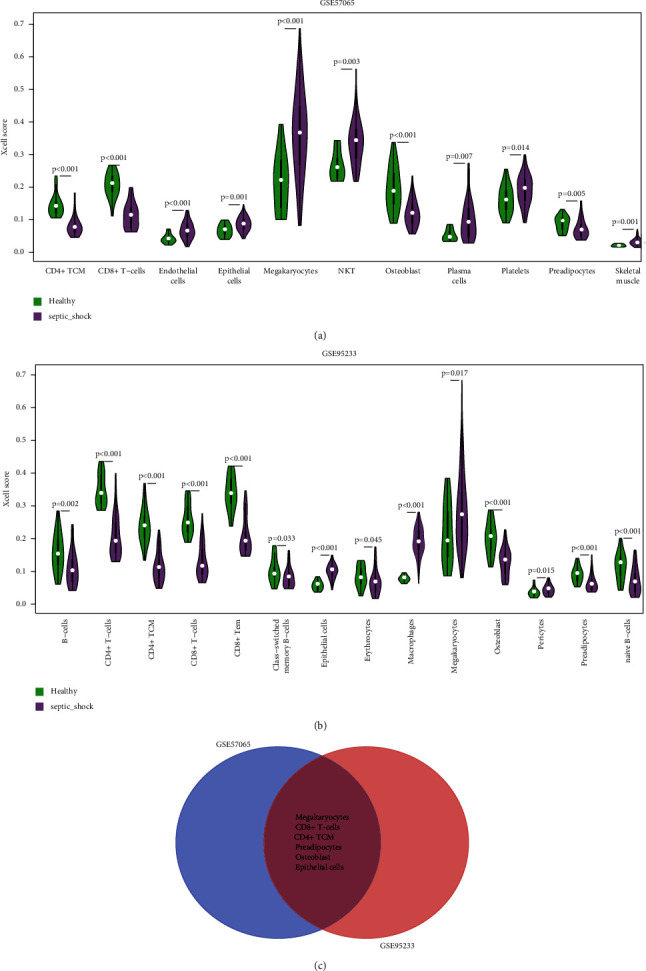
Differential analysis of immune and stromal cell enrichment. Violin plots show the immune and stromal cells with significant differences in enrichment scores between SS samples and controls in GSE57065 (a) and GSE95233 (b). (c) Venn diagram of the intersection of cells (megakaryocytes, CD8 + T-cells, CD4 + TCM, preadipocytes, osteoblasts, and epithelial cells) with significant differences in relative abundance between SS samples and controls in both datasets.

To evaluate differences in immune cell enrichment between the two groups, we obtained the enrichment scores for 64 types of immune cells and stromal cells in each sample and compared SS and healthy controls by a Wilcoxon test (Figures 6(a) and 6(b)). In total, 11 and 14 types of cells had significantly different enrichment scores between the two groups in GSE57065 and GSE95233, respectively. By considering the intersection (Figure 6(c)), six types of cells, including megakaryocytes, CD8 + T cells, CD4 + TCM, preadipocytes, osteoblasts, and epithelial cells, differed significantly between SS samples and controls in both datasets.

### 3.7. Correlations between Pathways and Immune Cells

The intersection of significant pathways obtained by GSEA and GSVA included 12 KEGG pathways. Spearman correlation coefficients were obtained to evaluate the relationships among these 12 key pathways and frequencies of six significant cell types in each sample. Accordingly, 41 and 27 cell-pathway relationships were obtained in GSE57065 and GSE95233, respectively. Finally, 21 overlapped cell-pathway relationships with significant positive correlations were identified. These 21 cell-pathway relationships contained 10 key pathways and 3 key cells, including CD8 + T cells, CD4 + T cells, and preadipocytes; their relationships in GSE57065 and GSE95233 are summarized in Table 1.

### 3.8. Predictive Performance of Key Genes

**Figure 7:**
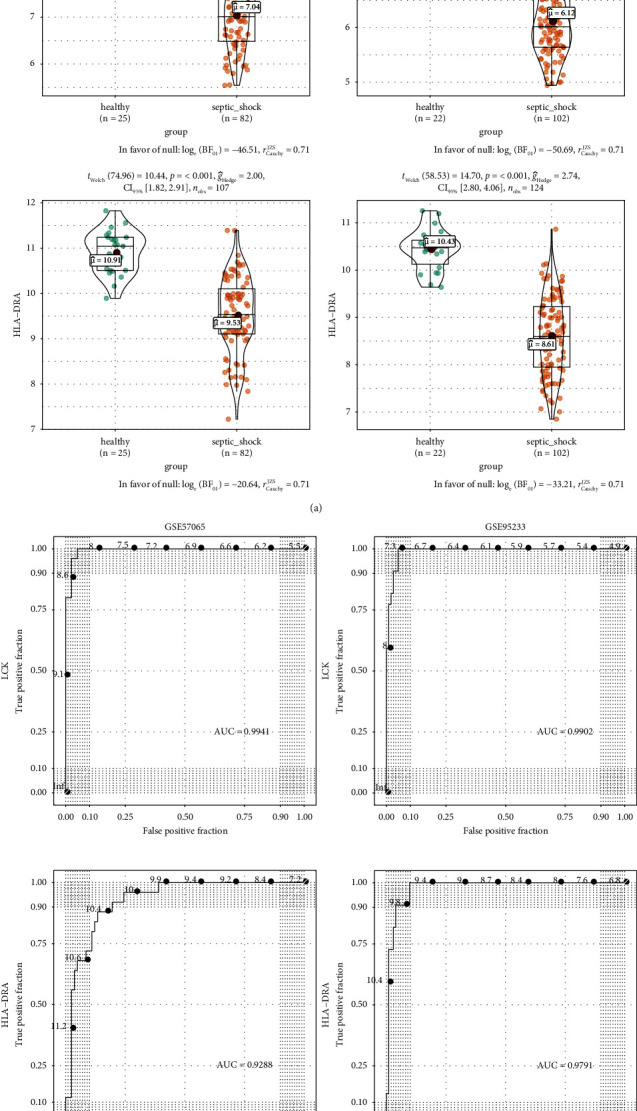
Predictive performance of key genes in the cell-pathway relations. (a) Violin plots show that *LCK* and *HLA-DRA*, which had the highest in the PPI network, were significantly differentially expressed between SS samples and controls in GSE57065 and GSE95233. (b) ROC curves suggest that *LCK* and *HLA-DRA* have excellent abilities to diagnose SS with AUCs >0.9.

The intersection of genes involved in 10 key pathways and genes in the PPI network (with degrees >10), and 17 key genes (*CD8A*, *HLA-DPA1*, *HLA-DPB1*, *HLA-DQA1*, *HLA-DQB1*, *HLA-DRA*, *ZAP70*, *MAPK14*, *CD247*, *CD3D*, *CD3E*, *CD3G*, *LCK*, *PRKCQ*, *ITK*, *LAT*, and *FYN*) were obtained, as shown in Table 2. The differences in these 17 genes between SS samples and controls in GSE57065 and GSE95233 were analyzed, and ROC curves were compared to explore their diagnostic value in SS. All 17 genes were significantly differentially expressed in the two datasets (*p* < 0.05). Among them, 16 genes were downregulated, and *MAPK14* was upregulated in SS. Furthermore, the ROC curves based on the two datasets suggested that these candidate genes had superior diagnostic values for SS, with area under the curve (AUC) values greater than 0.9. The violin plots and ROC curves for *LCK* and *HLA-DRA*, which had the highest degrees of connections in the PPI network, are shown in Figures 7(a) and 7(b).

### 3.9. Verification with External Datasets

**Figure 8:**
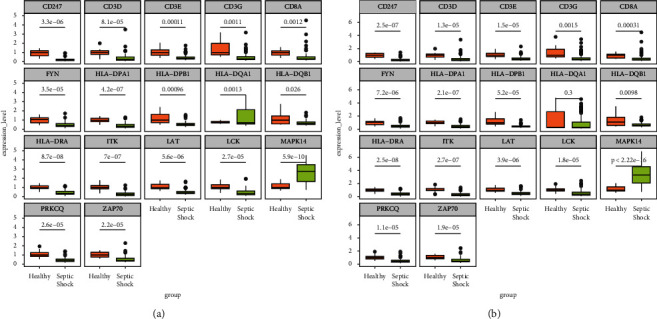
External expression verification of 17 candidate genes using the GSE4607 (a) and GSE13904 (b) datasets.

**Figure 9:**
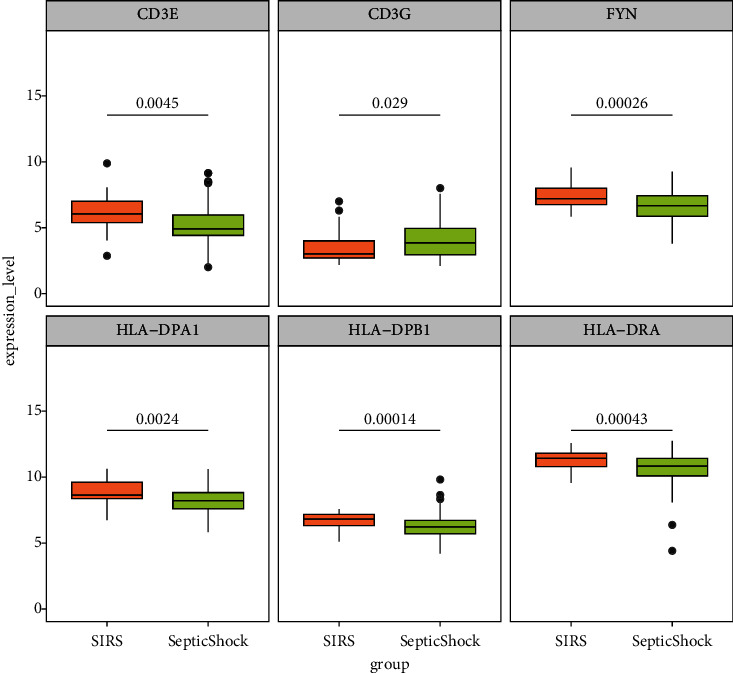
Differences in the expression of candidate genes between SIRS and SS samples from GSE66099.

To verify the expression differences in the 17 candidate genes between healthy controls and SS samples, GSE4607 and GSE13904 datasets were used for external verification. In the GSE4607 dataset (Figure 8(a)), all genes showed significant differences in expression between the two groups, of which only *MAPK14* was upregulated in SS. In the GSE13904 dataset (Figure 8(b)), *MAPK14* was still overexpressed in SS, and the other 15 genes were significantly downregulated in the SS samples, except *HLA−DQA1*. The GSE66099 dataset was subsequently included to verify whether these genes have expression specificity in SS that enables their distinction from other types of inflammation. Expression levels of *CD3E*, *CD3G*, *FYN*, *HLA-DPA1*, *HLA-DPB1*, and *HLA-DRA* differed significantly between SIRS and SS (Figure 9), indicating that these six genes may contribute to SS inflammation.

### 3.10. Experimental Expression Validation

**Figure 10:**
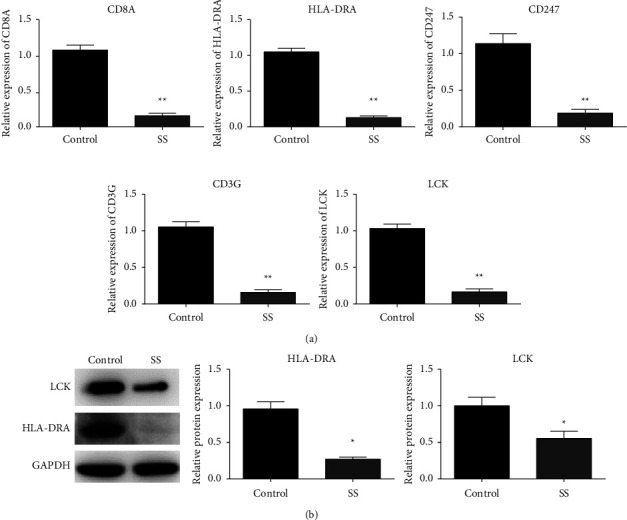
Validation of expression patterns of key genes. (a) qRT-PCR results suggest that the mRNA expression levels of *CD8A*, *CD247*, *CD3G*, *LCK*, and *HLA-DRA* were lower in SS samples than in control samples. (b) Western blotting results revealed that the expression levels of LCK and HLA-DRA were downregulated in SS at the protein level. ^*∗*^*p* < 0.05; ^*∗∗*^*p* < 0.01.

A total of 12 whole blood samples (6 cases and 6 controls) were collected to validate the expression of key genes. Among the 17 key genes, those with the highest degrees of connections in the PPI network (*LCK* and *HLA-DRA*) and genes with the highest fold change values in the GSE57065 or GSE95233 datasets (*CD8A*, *CD247*, and *CD3G*) were selected for validation by qRT-PCR. As depicted in Figure 10(a), the mRNA expression levels of these five genes were significantly lower in SS samples than in the control group. As determined by western blotting (Figure 10(b)), the protein expression levels of LCK and HLA-DRA were significantly lower in patients with SS than in healthy controls.

## 4. Discussion

Sepsis and SS are life-threatening diseases caused by a dysregulated immune response to infection and may lead to tissue and organ damage and even death [31, 32]. Currently, there is no effective treatment for SS. Accordingly, the disease burden can only be reduced by early detection, resuscitation, and the prompt administration of appropriate antibiotics [9]. Unlike sepsis, SS leads to uncontrolled and intensified inflammatory responses, but the exact timing of triggering this process is elusive, which underscores the importance of identifying gene expression differences between SS and normal controls, rather than sepsis, for diagnosis in patients early in disease progression [33]. Therefore, this study compared mRNA expression profiles between SS and normal samples and then identified 17 immune-related genes involved in the progression of SS by bioinformatics analyses. These genes also showed the potential to identify the risk of SS development in the validation cohorts. Five of these 17 genes (including *CD8A*, *CD247*, *CD3G*, *LCK*, and *HLA-DRA*) were experimentally validated for expression, considering their central roles in the PPI network and their consistent downregulation in SS samples were finally confirmed. The novel biomarkers proposed in this study are important to improve early identification and the management of acute episodes and to reduce septicemic deaths and disability. There are several similar articles that reported the diagnostic biomarkers for SS [34, 35], but our study differs in that we focus more on the gene set involved in SS-related immune regulation. Therefore, we also proposed three key immune cells including CD4 + T cells, CD8 + T cells, and preadipocytes that may be regulated by immune-related candidate genes and involved in disease progression in SS. These potential immune regulatory mechanisms are important clues for understanding the role of candidate genes in disease diagnosis and for developing new drugs to prevent SS.

In this study, DEGs between SS samples and controls were mainly enriched in biological functions and pathways related to immunological and inflammatory responses. Using the GSVA algorithm, significant differences in immune-related functions and pathways were also found between them, including the T-cell receptor signaling pathway, autoimmune disease, and primary immunodeficiency, among others. A related bioinformatics analysis supported our findings and demonstrated that DEGs in SS were involved in immune response, chemokine-mediated signaling, neutrophil chemotaxis, and chemokine activity [36]. Additionally, immunodeficiency is commonly observed in patients with severe sepsis and SS and is associated with an increased risk of short-term mortality [16].

Considering the role of immunodeficiency in SS development, we carried out deconvolution and correlation analyses to determine the effect of immune cells on SS. Based on our results, three key immune cell types, i.e., CD4 + T cells, CD8 + T cells, and preadipocytes, differed significantly between SS samples and controls. In terms of adaptive immunity, sepsis-induced apoptosis leads to lymphocytopenia in patients with SS, and this process involves all types of T cells, including *T* regulatory cells, CD4 + T cells, CD8 + T cells, and natural killer cells, which are conducive to immunosuppression [14]. Immunosuppression is a compensatory anti-inflammatory response that explains the short-term death of SS patients, while survivors may experience a prolonged state of immunosuppression, which could be reactivated by pathogenic infection [37]. During the immunosuppression in SS, the loss of T-cell function is associated with reduced resistance to secondary infections in patients with SS [38]. Furthermore, decreased expression of cytotoxic molecules weakens the lytic activity of CD8 + T cells [39]. In this study, the enrichment scores for CD4 + T cells and CD8 + T cells were found to be significantly lower in SS cases than in controls. Roger et al. further supported our findings and proposed that rates of CD4 + T cell and CD8 + T cell apoptosis were higher in patients with SS than in controls [40]. The above evidence indicated that DEGs identified in this study may trigger immunosuppression and lead to SS by down-regulating CD4 + T cell and CD8 + T cell levels. In addition, we also proposed a relationship between preadipocytes and immune deficiency and T-cell receptor-related pathways. Several factors secreted by preadipocytes have pro-inflammatory and anti-inflammatory effects and can contribute to diseases associated with immune system dysfunction [41]. Some immunodeficiency virus protease inhibitors inhibit preadipocyte differentiation and promote adipocyte death [42]. In this study, we found several genes involved in the T-cell receptor signaling pathway, of which *FYN* is expressed in human preadipocytes and is induced after the initiation of differentiation [43]. Therefore, we hypothesized that preadipocytes participate in the activation of immune-related pathways during SS development by inducing the expression of *FYN*; however, further mechanistic investigations are still needed.

Furthermore, 17 key genes were identified in immune-related cell-pathway pairs, and core genes from the PPI network were selected for expression validation. Relevant results suggested that the mRNA expression levels of *CD8A*, *CD247*, and *CD3G* were downregulated in SS samples, while *LCK* and *HLA-DRA* were decreased at both the mRNA and protein levels. As a member of the Src family of kinases, LCK is involved in changes in the activity of CD4 + T cells and CD8+ T cells during T-cell development [44]. *LCK* plays a crucial role in T-cell differentiation, survival, and activation [45]; however, the contribution of *LCK* to the development of SS has not been explored. With regard to *HLA-DRA*, studies have found that the reduced expression of *HLA-DR* mRNA is correlated with increased mortality after SS [46]. Winkler et al. reported that *HLA-DR* expression is decreased in sepsis [47], and the reduced *HLA-DR* expression may be a characteristic feature of septic monocytes [14]. Furthermore, the dynamic changes in HLA-DRA gene expression and helper T cell subsets in patients with sepsis are indicative of immunosuppression [48]. Combined with the results of this study, we speculated that the loss of LCK and HLA-DRA expression may lead to the failure of T-cell differentiation and dynamic changes of helper T cell subsets, thus resulting in immunosuppression and the onset of SS.

The main limitation of this study is that patients with autoimmune diseases were excluded from the independent clinical validation cohort, which to some extent affects the extrapolation of the results. Furthermore, we only performed a preliminary exploration of the potential roles of these genes in disease. The regulatory mechanisms by which these candidate genes contribute to immunosuppression during SS are not clearly established. In future studies, bioinformatic analyses will be used to predict the upstream regulatory mechanisms, and experimental approaches will also be carried out to confirm the regulatory effects of the candidate genes. Additionally, due to limited sample size, we did not further screen the most robust biomarkers out from the 17 identified key genes by LASSO regression and/or multivariate Cox analyses. In the future, a most valuable predictive signature should be developed based on more convincing methods and larger sample size.

In conclusion, we found that DEGs between SS cases and controls were mainly enriched in immune- and inflammation-related functions and pathways. In addition, CD4 +  T cells, CD8 + T cells, and preadipocytes were proposed as key immune cells to involve in the SS progression. These immune cells were also associated with 17 key immune-related genes, among which the downregulation of *CD8A*, *CD247*, *CD3G*, *LCK*, and *HLA-DRA* in SS samples was further experimentally validated. Our findings reveal several novel biomarkers for the early identification of SS.

## Figures and Tables

**Table 1:** Correlations between KEGG pathway enrichment score and deconvolution scores for key cells in GSE57065 and GSE95233.

Pathway	Cell type	GSE57065			GSE95233		
		*r*	95% CI	*p*	*r*	95%CI	*p*
KEGG_ALLOGRAFT_REJECTION	CD4 + TCM	0.56	0.42–0.68	3.26*E* − 10	0.62	0.50–0.72	1.37*E* − 14
KEGG_ALLOGRAFT_REJECTION	CD8 + T-cells	0.77	0.68–0.84	6.62*E* − 22	0.62	0.50–0.72	1.66*E* − 14
KEGG_ANTIGEN_PROCESSING_AND_PRESENTATION	CD4 + TCM	0.41	0.26–0.57	1.45*E* − 05	0.61	0.49–0.71	3.16*E* − 14
KEGG_ANTIGEN_PROCESSING_AND_PRESENTATION	CD8 + T-cells	0.73	0.64–0.82	4.55*E* − 19	0.75	0.66–0.82	1.07*E* − 23
KEGG_AUTOIMMUNE_THYROID_DISEASE	CD4 + TCM	0.47	0.30–0.60	4.03*E* − 07	0.62	0.50–0.72	1.37*E* − 14
KEGG_AUTOIMMUNE_THYROID_DISEASE	CD8 + T-cells	0.60	0.46–0.71	8.79*E* − 12	0.62	0.50–0.72	1.66*E* − 14
KEGG_CELL_ADHESION_MOLECULES_CAMS	CD4 + TCM	0.55	0.37–0.65	7.21*E* − 10	0.61	0.49–0.71	3.16*E* − 14
KEGG_CELL_ADHESION_MOLECULES_CAMS	CD8 + T-cells	0.41	0.24–0.55	1.43*E* − 05	0.75	0.66–0.82	1.07*E* − 23
KEGG_GRAFT_VERSUS_HOST_DISEASE	CD4 + TCM	0.54	0.41–0.67	1.34*E* − 09	0.62	0.50–0.72	1.37*E* − 14
KEGG_GRAFT_VERSUS_HOST_DISEASE	CD8 + T-cells	0.76	0.68–0.84	2.65*E* − 21	0.62	0.50–0.72	1.66*E* − 14
KEGG_INTESTINAL_IMMUNE_NETWORK_FOR_IGA_PRODUCTION	CD4 + TCM	0.69	0.60–0.79	1.35*E* − 16	0.59	0.46–0.70	4.34*E* − 13
KEGG_INTESTINAL_IMMUNE_NETWORK_FOR_IGA_PRODUCTION	CD8 + T-cells	0.74	0.66–0.83	1.08*E* − 19	0.50	0.35–0.62	4.56*E* − 09
KEGG_PRIMARY_IMMUNODEFICIENCY	CD4 + TCM	0.52	0.38–0.66	7.43*E* − 09	0.68	0.57–0.76	7.65*E* − 18
KEGG_PRIMARY_IMMUNODEFICIENCY	CD8 + T-cells	0.83	0.77–0.89	3.70*E* − 28	0.88	0.83–0.92	1.75*E* − 41
KEGG_PRIMARY_IMMUNODEFICIENCY	Preadipocytes	0.65	0.52–0.74	2.71*E* − 14	0.56	0.43–0.67	1.26*E* − 11
KEGG_T_CELL_RECEPTOR_SIGNALING_PATHWAY	CD8 + T-cells	0.70	0.57–0.78	9.41*E* − 17	0.87	0.82–0.91	1.08*E* − 39
KEGG_T_CELL_RECEPTOR_SIGNALING_PATHWAY	Preadipocytes	0.58	0.39–0.66	5.64*E* − 11	0.48	0.33–0.60	2.20*E* − 08
KEGG_TYPE_I_DIABETES_MELLITUS	CD4 + TCM	0.52	0.37–0.65	9.03*E* − 09	0.62	0.50–0.72	1.37*E* − 14
KEGG_TYPE_I_DIABETES_MELLITUS	CD8 + T-cells	0.72	0.62–0.80	3.83*E* − 18	0.62	0.50–0.72	1.66*E* − 14
KEGG_VIRAL_MYOCARDITIS	CD4 + TCM	0.43	0.28–0.58	2.86*E* − 06	0.62	0.50–0.72	1.37*E* − 14
KEGG_VIRAL_MYOCARDITIS	CD8 + T-cells	0.55	0.42–0.68	7.63*E* − 10	0.62	0.50–0.72	1.66*E* − 14

KEGG, Kyoto Encyclopedia of Genes and Genomes; CI, confidence interval. *r* indicates the Spearman correlation coefficient. *p* < 0.05 indicates statistical significance.

**Table 2:** Key genes in enriched KEGG pathways.

Pathways	Genes
KEGG_CELL_ADHESION_MOLECULES_CAMS	*CD8A; HLA-DPA1; HLA-DPB1; HLA-DQA1; HLA-DQB1; HLA-DRA*
KEGG_ANTIGEN_PROCESSING_AND_PRESENTATION	*CD8A; HLA-DPA1; HLA-DPB1; HLA-DQA1; HLA-DQB1; HLA-DRA*
KEGG_T_CELL_RECEPTOR_SIGNALING_PATHWAY	*ZAP70; MAPK14; CD8A; CD247; CD3D; CD3E; CD3G; LCK; PRKCQ; ITK; LAT; FYN; HLA-DRA*
KEGG_INTESTINAL_IMMUNE_NETWORK_FOR_IGA_PRODUCTION	*HLA-DRA*
KEGG_TYPE_I_DIABETES_MELLITUS	*HLA-DPA1; HLA-DPB1; HLA-DQA1; HLA-DQB1; HLA-DRA*
KEGG_AUTOIMMUNE_THYROID_DISEASE	*HLA-DPA1; HLA-DPB1; HLA-DQA1; HLA-DQB1; HLA-DRA*
KEGG_ALLOGRAFT_REJECTION	*HLA-DPA1; HLA-DPB1; HLA-DQA1; HLA-DQB1; HLA-DRA*
KEGG_GRAFT_VERSUS_HOST_DISEASE	*HLA-DPA1; HLA-DPB1; HLA-DQA1; HLA-DQB1; HLA-DRA*
KEGG_PRIMARY_IMMUNODEFICIENCY	*ZAP70; CD8A; CD3D; CD3E; LCK*
KEGG_VIRAL_MYOCARDITIS	*HLA-DPA1; HLA-DPB1;HLA-DQA1;HLA-DQB1;FYN;HLA-DRA*

KEGG, Kyoto Encyclopedia of Genes and Genomes.

## Data Availability

Microarray datasets including GSE57065, GSE95233, GSE4607, GSE13904, and GSE66099 used in this study can be downloaded from the GEO database at http://www.ncbi.nlm.nih.gov/geo/. The raw data of qRT-PCR and western blot are available at https://doi.org/10.4121/19074482.

## Supplementary Materials

Supplementary Materials Figure S1. Study flowchart. Table S1. The sequences of primers used for qRT-PCR.
