# Supplementary material for: Identification of Potential Biomarkers of Septic Shock Based on Pathway and Transcriptome Analyses of Immune-Related Genes
Source: Genet Res (Camb). 2023 Aug 5;2023:9991613. doi: 10.1155/2023/9991613 (PMC10423089; doi:10.1155/2023/9991613)
Supplement: Supplementary Materials — Figure S1. Study flowchart. Table S1. The sequences of primers used for qRT-PCR. [file 9991613.f1.zip › Supplementary Table S1.docx]

**Table S1.** The sequences of primers used for qRT-PCR

| **Gene** | **Sequences (5’-3’)** |
| --- | --- |
| CD8A-hF | GCTGGACTTCGCCTGTGATA |
| CD8A-hR | GGGCTTGTCTCCCGATTTGA |
| HLA-DRA-hF | TCACGTGGCTTCGAAATGGA |
| HLA-DRA-hR | TCCACCCTGCAGTCGTAAAC |
| CD247-hF | TTGCCGATTACAGAGGCACAG |
| CD247-hR | GCCACGTCTCTTGTCCAAAAC |
| CD3G-hF | GGCTGTCCTCATCCTGGCTAT |
| CD3G-hR | CGAGGGTCCTTGGCATTACTT |
| LCK-hF | GCTGGTTCGGCTCTACGCT |
| LCK-hR | GCCATGTCCAGGAGTTTGTTG |
| GAPDH-hF | TGACAACTTTGGTATCGTGGAAGG |
| GAPDH-hR | AGGCAGGGATGATGTTCTGGAGAG |

F: forward; R: reserve.
