# Supplementary figures and images for: Identification of Potential Biomarkers of Septic Shock Based on Pathway and Transcriptome Analyses of Immune-Related Genes
Source: Genet Res (Camb). 2023 Aug 5;2023:9991613. doi: 10.1155/2023/9991613 (PMC10423089; doi:10.1155/2023/9991613)

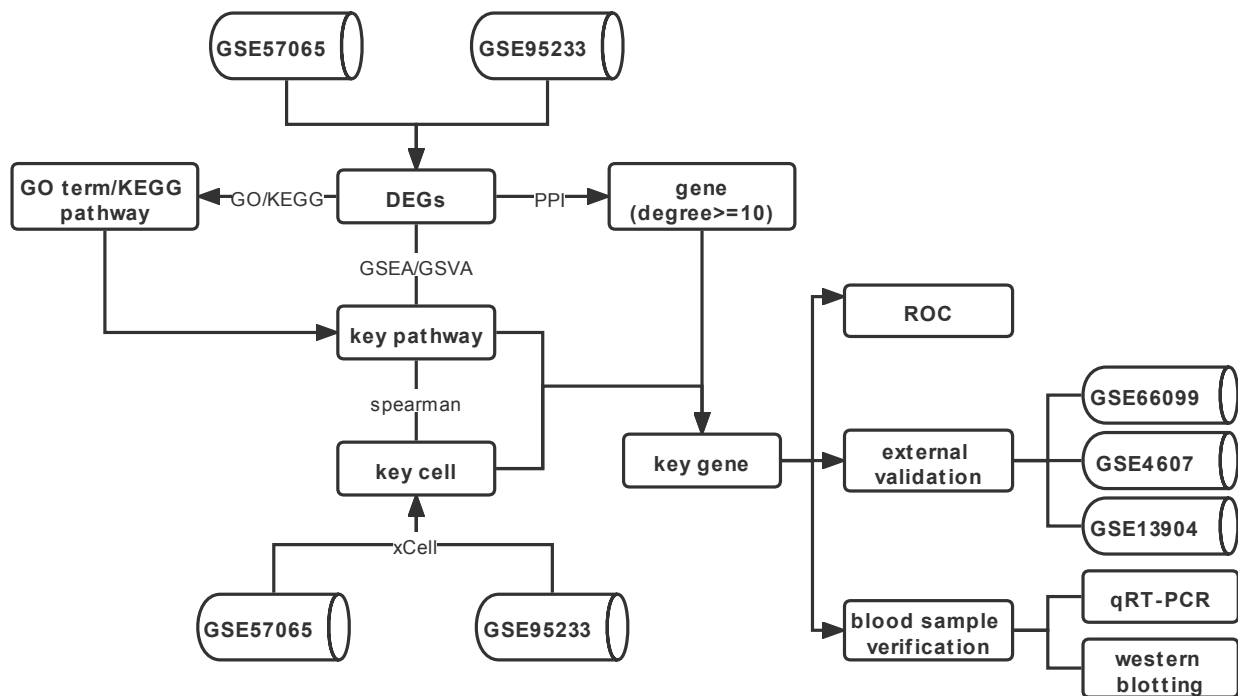

Supplement: Supplementary Materials — Figure S1. Study flowchart. Table S1. The sequences of primers used for qRT-PCR. [file 9991613.f1.zip › Supplementary Figure S1.pdf]
